# Melatonin and plants

**DOI:** 10.1093/jxb/eru523

**Published:** 2015-01-06

**Authors:** Dun-Xian Tan

**Affiliations:** The University of Texas, Health Science Center at San Antonio, TX, USA

Melatonin was initially considered as a signalling molecule only occurring in animals, but as further evidence emerged, it was recognized that it is a phylogenetically ancient molecule. Its presence can be traced to primitive photosynthetic bacteria, red and green algae, fungi, and plants. From an evolutionary point of view, a primary function of this molecule is as a free radical scavenger and antioxidant to protect organisms from environmental and internal oxidative stresses. Other functions were acquired during evolution (Tan *et al.*, 2014). Melatonin was found in plants two decades ago (Hattori *et al.*, 1995), and since then studies have focused primarily on its medicinal and nutritional importance, in both animals and humans, with little attention given to potential physiological and biological roles in the plants themselves. During recent years, however, identifying the functions of melatonin in plants has become a rapidly progressing field, and this is partially reflected in this special issue of the *Journal of Experimental Botany*, ‘Melatonin and Plants’. In this issue, Hardeland (2015) reviews, mainly from an evolutionary point of view, the occurrence and functions of melatonin in phototrophs, and the place that melatonin deserves in the network of phytohormones.

Melatonin not only regulates the germination, growth, and reproduction of plants (Tan *et al.*, 2012), but also promotes the ripening of fruit. Sun *et al.* (2015) report that melatonin upregulates the expression of ethylene signal transduction-related genes and enhances ethylene biosynthesis, perception, and signalling, thereby promoting postharvest ripening and improvement of the quality of tomato fruit. Most importantly, melatonin is an essential molecule for protecting plants from abiotic and biotic stressors. Many studies show that exogenously applied or endogenously produced melatonin significantly enhances the tolerance of plants to abiotic stressors including hot or cold temperatures, salinity, drought, over-watering, ultraviolet radiation, and chemical or metal pollutants in water and soils. The molecular and metabolic mechanisms by which melatonin enhances the tolerance of plants to drought and salinity have been studied and are reported in this collection. Li *et al.* (2015) have observed that in pretreated apple leaves under drought stress, melatonin application downregulates gene expression of the abscisic acid (ABA) pathway, decreasing the ABA content while maintaining open stomata. Shi *et al.* (2015) found that genes involved in nitrogen metabolism, major carbohydrate metabolism, the tricarboxylic acid/org transformation, transport, hormone metabolism, metal handling, redox reactions, and secondary metabolism are upregulated after melatonin pre-treatment in bermudagrass cultivars; thus, melatonin enhances the tolerance of the plants to multiple abiotic stresses including drought. Using soybean as an experimental plant, Wei *et al.* (2015) showed that melatonin application during germination significantly promotes soybean growth and seed production, and also enhances its tolerance to salt stress by modifying the oxidoreductase activity/process as well as secondary metabolic processes. In addition, an excellent review by Zhang *et al.* (2015) discuss the mechanisms by which melatonin alleviates abiotic stresses

In addition to enhancing tolerance to abiotic stress, melatonin has also recently been shown to protect plants against biotic stress (Yin *et al.*, 2013; Lee *et al.*, 2014). The protective mechanisms involve free radical scavenging, upregulation of an array of stress response genes, enhancing the activities of several catabolic enzymes, and preserving the integrity and functions of chloroplasts under stressful conditions. As a protective molecule, an advantage of melatonin in plants is that it is stress inducible. Gene expression of melatonin synthetic enzymes, particularly the rate-limiting enzyme serotonin *N*-acetyltransferase (SNAT), is significantly upregulated in response to stress (Byeon and Back, 2014). Thus, the production of melatonin in plants is always in parallel with the degree of the overriding stressors. This feature makes melatonin highly efficient as a stress protector.

Chloroplasts are believed to be a primary site of melatonin production. The SNAT has been localized in chloroplasts of the rice plant (Byeon *et al.*, 2014). This is not surprising, since cyanobacteria are considered to be the precursors of chloroplasts, and these organisms already have the capacity to synthesize melatonin (Tan *et al.*, 2013). In this collection, Byeon *et al.* (2015) report that chloroplasts of red algae encode the melatonin synthetic enzyme gene, *SNAT*. This observation provides direct evidence that melatonin synthesis occurs in the chloroplast.

The chloroplast is a specialized organelle for photosynthesis in plants. During photosynthesis a large quantity of reactive oxygen species (ROS) are produced and locally generated melatonin would provide on-site protection against these toxic ROS. Melatonin has been observed to preserve chlorophyll and improve the photosynthetic efficiency of chloroplasts in plants under stress. Of course, additional underlying mechanisms to explain melatonin’s high efficiency in enhancing the tolerance of plants to environmental stresses will probably be uncovered. It is my hope that the present collection of papers on melatonin and plants will stimulate further research in this new and rapidly developing field.
